# *In Situ* OH Generation from O_2_^−^ and H_2_O_2_ Plays a Critical Role in Plasma-Induced Cell Death

**DOI:** 10.1371/journal.pone.0128205

**Published:** 2015-06-05

**Authors:** Dehui Xu, Dingxing Liu, Biqing Wang, Chen Chen, Zeyu Chen, Dong Li, Yanjie Yang, Hailan Chen, Michael G. Kong

**Affiliations:** 1 Centre for Plasma Biomedicine, State Key Laboratory of Electrical Insulation and Power Equipment, Xi’an Jiaotong University, Xi’an, China; 2 Frank Reidy Center for Bioelectrics, Old Dominion University, Norfolk, United States of America; 3 Department of Electrical and Computer Engineering, Old Dominion University, Norfolk, United States of America; University Paul Sabatier, FRANCE

## Abstract

Reactive oxygen and nitrogen species produced by cold atmospheric plasma (CAP) are considered to be the most important species for biomedical applications, including cancer treatment. However, it is not known which species exert the greatest biological effects, and the nature of their interactions with tumor cells remains ill-defined. These questions were addressed in the present study by exposing human mesenchymal stromal and LP-1 cells to reactive oxygen and nitrogen species produced by CAP and evaluating cell viability. Superoxide anion (O_2_^−^) and hydrogen peroxide (H_2_O_2_) were the two major species present in plasma, but their respective concentrations were not sufficient to cause cell death when used in isolation; however, in the presence of iron, both species enhanced the cell death-inducing effects of plasma. We propose that iron containing proteins in cells catalyze O_2_^−^ and H_2_O_2_ into the highly reactive OH radical that can induce cell death. The results demonstrate how reactive species are transferred to liquid and converted into the OH radical to mediate cytotoxicity and provide mechanistic insight into the molecular mechanisms underlying tumor cell death by plasma treatment.

## Introduction

Cold atmospheric plasma (CAP), an ionized gas, has many biological applications including wound healing, surgical procedures, disinfection, and even cancer treatment[1–5]. Dielectric barrier discharge (DBD) and the plasma jet are two methods for producing CAP; both generate various kinds of reactive oxygen and nitrogen species (ROS and RNS, respectively), including hydroxyl radical (OH), hydrogen peroxide (H_2_O_2_), ozone (O_3_), atomic oxygen (O), superoxide anion (O_2_^−^), nitric oxide (NO), and peroxynitrite anion (ONOO^−^) [6], which are considered as the most biologically relevant components of plasma. Reactive species composition in CAP can be altered by regulating the voltage, frequency, working and feeding gases, and humidity. While many studies have shown that CAP is an efficient disinfectant and can also kill normal as well as tumor cells [7–9], it remains unclear which reactive species are chiefly responsible for these biological effects.

Since tissues and cells are immersed in liquid, studies have mostly focused on the interaction of the plasma with a liquid medium. ROS and RNS undergo conversion into different types of reactive species when transferred from gas to liquid phase. Our previous work showed that O_2_^−^ and H_2_O_2_ can permeate in distilled water to a greater extent than other species and may interact with cellular components [10]. Computer simulations have shown that OH, HO_2_, and H_2_O_2_ can travel deep into a liquid layer to reach biomolecules [11], and that O_2_^−^, ONOO^−^, NO_3_^−^, O_3_, H_2_O_2_, and HNO_X_ are the predominant species generated after treatment of a 50–400 μM thick water layer with a DBD plasma device [12]. Another study that measured O_2_^−^ and OH using the spin trapping compound CYPMPO and detected the signal by electron spin resonance (ESR) spectrometry showed that O_2_^−^ and OH density varied according to plasma jet settings, although the range of concentrations was not reported [13]. OH radicals in 3 ml of aqueous solution produced by atmospheric-pressure He plasma jet measured using terephthalic acid (TA) as a spin trapping compound were present at a concentration of 3.3 uM [14]. The concentration of OH and O_2_^−^ in various ionic solutions was about 1–10 μM after a 3 min Ar plasma treatment, as measured by ESR [15]. However, there is no information about whether these species can interact with biomolecules in liquid medium during plasma treatment.

The present study investigated this question in human mesenchymal stromal cells (MSCs) and LP-1 myeloma tumor cells exposed to CAP generated by a plasma jet. The results demonstrate that O_2_^−^ and H_2_O_2_ are the two major reactive species in liquid but are present at concentrations that are insufficient to cause cell death; this was ultimately induced by the OH radical generated *in situ* upon exposure of cells to O_2_^−^ and H_2_O_2_ in the plasma. These findings provide insight into the molecular mechanisms underlying plasma-induced tumor cell death, and may also provide a basis for generating a more powerful plasma enriched with particular reactive species for biological applications such as cancer treatment.

## Materials and Methods

### Plasma generation and characterization

**Fig 1:**
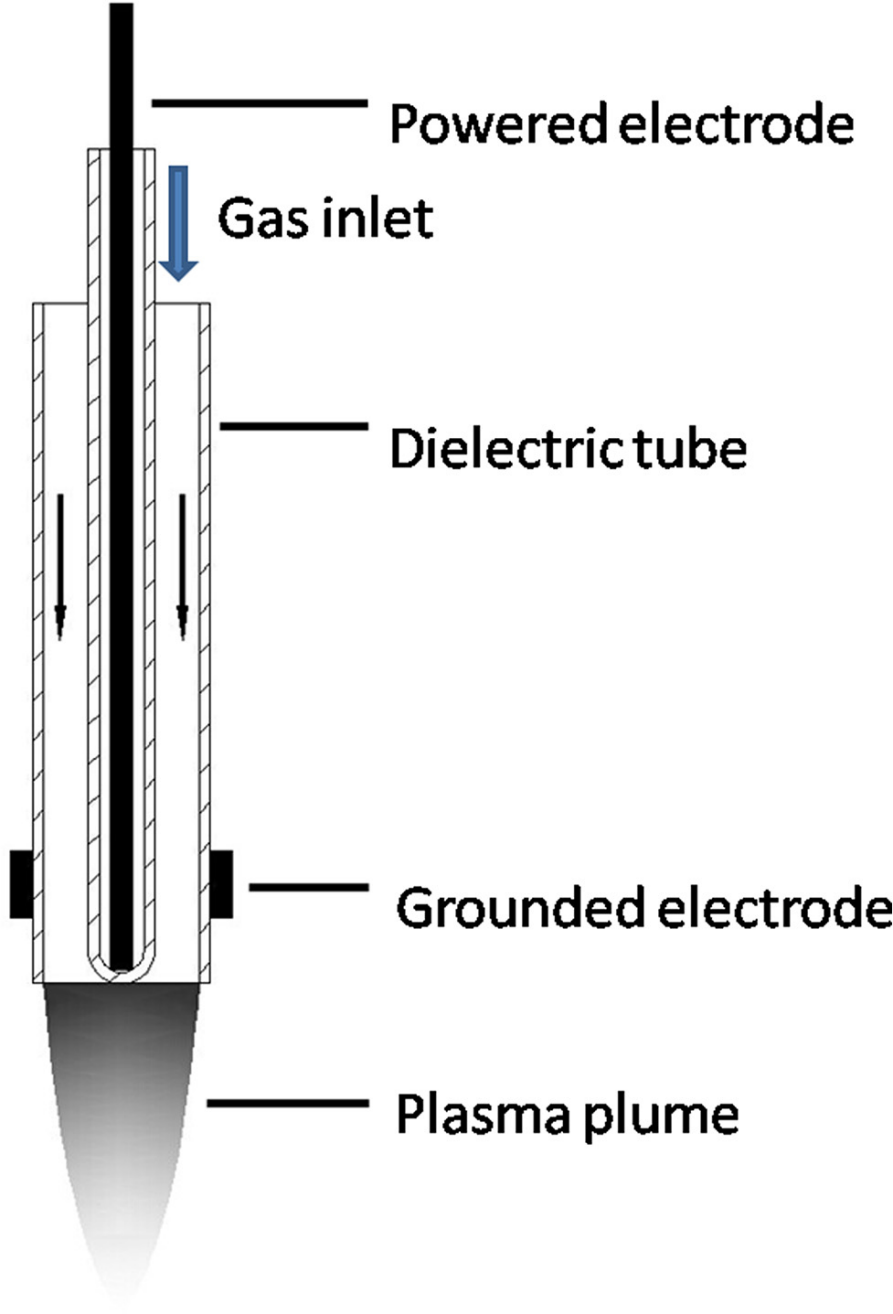
Schematic illustration of the plasma jet used in this study.

CAP was generated by a plasma jet system consisting of a 1 mm powered electrode enclosed in a quartz tube, with a grounded outer electrode wrapped around a 6.0 mm diameter dielectric tube (Fig 1). The system also included a gas flow controller, high-voltage power supply, oscilloscope, and plasma jet. A gas flow of 2 slm for He/Ar was used at voltages of 10 kHz/8 kV for He and 10 kHz/10 kV for Ar. The detailed experimental setup is shown in S1 Fig.

### Cell culture

Human MSCs and LP-1 cells [16] were cultured in Roswell Park Memorial Institute (RPMI)1640 medium supplemented with 10% fetal calf serum, 100 U/ml penicillin, 50 µg/ml streptomycin, and 2 mM l-glutamine (all from Hyclone, Logan, UT, USA), in an atmosphere of 5% CO_2_ at 37°C. MSCs were trypsinized and refreshed weekly and only cells from nine or fewer passages were used in the experiments.

### Plasma treatment

Normally, cells were cultured in a 24 well plate in 300 ul RPMI1640 medium at a concentration of 2 × 10^5^ cells. For cell viability assay, MSC cells were cultured in a 96 well plate in 100 ul RPMI1640 medium at a concentration of 1 × 10^5^ cells. Luminescence was measured 24 h after plasma treatment by directly adding 100 ul of Cell-Titer-Glo reagent into the cells, which could avoid trypsinization of MSC cells and losing some un-adherent cells after plasma treatment.

### Reagents

Radical scavengers were purchased from Sigma (St. Louis, MO, USA) [17]. The scavengers used were as follows: sodium pyruvate (10 mM) for H_2_O_2_ [18]; mannitol (50 mM) for OH [18]; carboxy-PTIO (100 μM) for NO [18]; trolox (100 μM) for peroxyl radical (ROO•) [19]; uric acid (100 μM) for O_3_ and ONOO^—^[18, 20]; sodium azide (1 mM) for singlet oxygen (^1^O_2_) [18]; and tiron (10 mM) for O_2_^–^ [21]. These are specific scavengers with little cross-reactivity to other ROS and are widely used for investigating the function of particular ROS [17]. Fe(III)-ethylenediaminetetraacetic acid (EDTA; 1 μM) was purchased from Tokyo Chemical Industry Co. Ltd. (Tokyo, Japan). Apo- and holo-ferritin were purchased from MP Biomedicals (Santa Ana, CA, USA).

### Cell viability assay

MSCs were seeded in RPMI1640 medium in 96 well optical plates at a concentration of 10^5^cells/100 ul well. After plasma treatment for 30 s, or 1 or 2 min, cells were grown for further 24 h. Cell viability was assessed using the CellTiter-Glo assay (Promega, Madison, WI, USA) according to the manufacturer’s instructions. CellTiter-Glo measures luminescence to quantify the level of ATP, which is positively correlated with cell viability. Briefly, 100 ul reagent were added to 100 ul cells and the mixture was lysed by placing on an orbital shaker for 2 min, followed by a 10 min incubation at room temperature. Luminescence was measured with a microplate reader (Varioskan Flash; Thermo Scientific, Waltham, MA, USA).

### Observation of morphological changes by microscopy

Cell morphological changes after plasma treatment were examined and imaged using an IX51 inverted phase contrast light microscope (Olympus, Tokyo, Japan). MSCs were normally adherent, with a polygonal shape; dying cells became shrunken and rounded, and detached from the plate.

### Flow cytometry for analysis of apoptosis

Cells were washed twice with Dulbecco’s Phosphate-Buffered Saline without calcium and magnesium (Ca^2+^/Mg^2+^-free DPBS; Corning, NY, USA) containing 0.5% bovine serum albumin and resuspended in 50 μl of 1× binding buffer (0.01 M Hepes/NaOH (pH 7.4), 0.14 M NaCl and 2.5 mM CaCl_2_) with 2.0 μl annexin V-fluorescein isothiocyanate (FITC) and 2.0 ul propidium iodide (PI) (Becton Dickinson, Franklin Lakes, NJ, USA) and incubated in the dark at room temperature for 15 min. An additional 400 μl of binding buffer were added and fluorescence was analyzed on an Accuri C6 flow cytometry (Becton Dickinson).

### H_2_O_2_ assay

H_2_O_2_ level was measured using the Amplex Red Hydrogen Peroxide Assay Kit (Invitrogen, Carlsbad, CA, USA). A working solution of 100 μM Amplex Red reagent and 0.2 U/ml horseradish peroxidase (HRP) was prepared beforehand. An H_2_O_2_ standard curve was generated with freshly prepared H_2_O_2_ (Invitrogen) concentrations ranging from 0 to 40 μM. A 50 μl volume of Amplex Red reagent/HRP working solution was added to each microplate well containing standards, controls, and samples (in a volume of 50 μl). The mixture was incubated at room temperature for 30 min while shielded from light. The H_2_O_2_ concentration was detected using a microplate reader (Thermo Scientific Varioskan Flash) at excitation and emission wavelengths of 530–560 nm and ~590 nm, respectively.

### Western blotting

Cell pellets were lysed in lysis buffer containing 50 mM Tris, 150 mM NaCl, 1% Nonidet P40, and 0.25% sodium deoxycholate. Cell debris was removed by centrifugation for 5 min at 14,000 rpm before sample buffer was added. After boiling, samples were separated by sodium dodecyl sulfate-polyacrylamide gel electrophoresis (SDS-PAGE) and transferred to polyvinylidene difluoride membranes (Bio-Rad, Hercules, CA, USA), which were blocked with PBS containing 5% low-fat milk and 0.1% Tween 20. Membranes were probed with antibodies against human ferritin heavy chain (FTH1) (1:1000) and β-actin (1:1000) (Cell Signaling Technology, Danvers, MA, USA). Membranes were washed with PBS containing 0.1% Tween 20 (PBST) for 30 min and then incubated with HRP-conjugated goat anti-rabbit IgG (1:2000 for FTHI) and anti-mouse IgG (1:2000 for β-actin) for 30 min at room temperature. Membranes were washed in PBST and imaged using a ChemiDoc-It 510 system (UVP, Upland, CA, USA).

### Coomassie Blue staining

Holo- and apo-transferrin were dissolved at concentrations of 0.1 mg/ml in 300 μl Ca^2+^/Mg^2+^-free DPBS and treated with Ar plasma for 2 or 8 min. Buffer was added and the samples were boiled, then separated by 12% SDS-PAGE and stained with Coomassie Blue for 20 min, followed by destaining for 1 h.

### Transfection of MSCs with pre-microRNA (miR)-200b

MSCs were cultured in a 6 well plate at 5 × 10^5^ cells/well in 2 ml RPMI1640 medium. Cells were transfected with pre-miR-200b (50 nM) using Lipofectamine 2000 (both from Invitrogen) according to the manufacturer’s instructions. Cells transfected with scrambled RNA oligonucleotide served as a control. Cells were harvested 48 h after transfection for western blot analysis. For the cell viability assay, cells were cultured in a 96 well plate at 10^5^ cells/well in 100 ul RPMI1640 medium and transfected with pre-miR-200b (50 nM) and scrambled control miRNA. After 24 h, cells were treated with Ar plasma for 20 s and cell viability was assessed with the CellTiter-Glo assay 24 h and 48 h later.

### Statistical analysis

All experimental conditions were prepared in triplicate and experiments were repeated at least three times. Data are presented as mean ± SD. Differences between groups were evaluated using the Mann-Whitney U test. P < 0.05 was considered statistically significant.

## Results and Discussion

**Fig 2:**
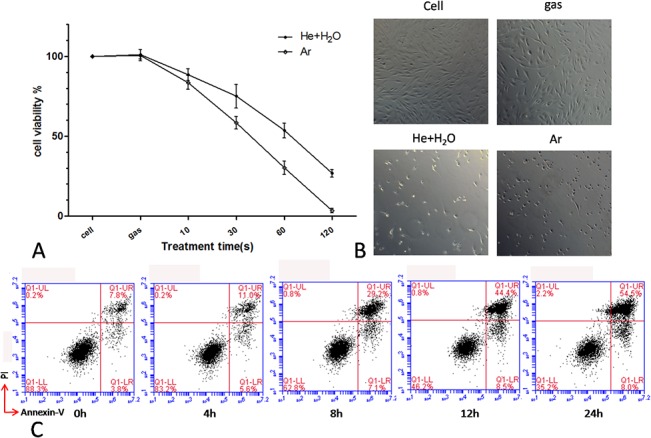
Cell death induced by He + H_2_O and Ar plasma. (A) Viability of MSCs 24 h after treatment with plasma for the indicated times. (B) Morphological changes in MSCs 24 h after treatment with plasma for 120 s. Ar gas flow without discharge was used as control. (C) Time course of MSC cell apoptosis by flow cytometry after Ar plasma treatment for 60 s.

A 2 slm gas flow was used to produce CAP by plasma jet. Two types of plasma—He + H_2_O and Ar—were tested. He + H_2_O plasma was produced at 10 kHz/8kV with 1% H_2_O in He gas (1.5 slm dry He gas with 0.5 slm humid He gas), while Ar plasma was produced at 10 kHz/10 kV. The distance between the plasma jet and liquid was fixed at 1.5 cm. The 2 slm of plasma gas flow resulted in slight evaporation of the 300 μl volume of medium in the 24 well plate, which was reduced by about 5 μl after plasma treatment for 2 min (data not shown). Next, MSCs cultured in 100 ul RPMI1640 medium in a 96 well plate were treated with plasma for various times (10, 30, 60, and 120 s) and cell viability was measured after 24 h. He + H_2_O and Ar plasma induced MSC apoptosis in a time-dependent manner (Fig 2A); this was accompanied by morphological changes after 120 s of plasma treatment, with cells gradually shrinking and becoming rounded (Fig 2B). A time series detection by flow cytometry of annexin-V and PI-stained cells showed that the number of annexin-V^+^ cells gradually increased over 24 h following a 60 s Ar plasma treatment (Fig 2C).

To evaluate the interaction between plasma and liquid, a computer simulation was used to calculate the distribution of different radicals in liquid water with the following set of equations.

$$\{\begin{array}{c} \frac{\partial c_{i}}{\partial t}+\frac{\partial \Gamma_{i}}{\partial x}=\frac{\partial c_{i}}{\partial t}-\frac{\partial }{\partial x}(D_{i,aq}\frac{\partial c_{i}}{\partial x})=\sum R_{i}(t)\\ \frac{\partial c_{j}}{\partial t}+\frac{\partial \Gamma_{j}}{\partial x}=\frac{\partial c_{j}}{\partial t}-\frac{\partial }{\partial x}(D_{j,aq}\frac{\partial c_{j}}{\partial x}\mp E_{x}c_{j}U_{j})=\sum R_{j}(t)\\ \frac{\partial^{2}V}{\partial x^{2}}=-\frac{\sum \rho_{net}}{\varepsilon_{H_{2}O}},E=-\frac{\partial V}{\partial x} \end{array}$$

**Fig 3:**
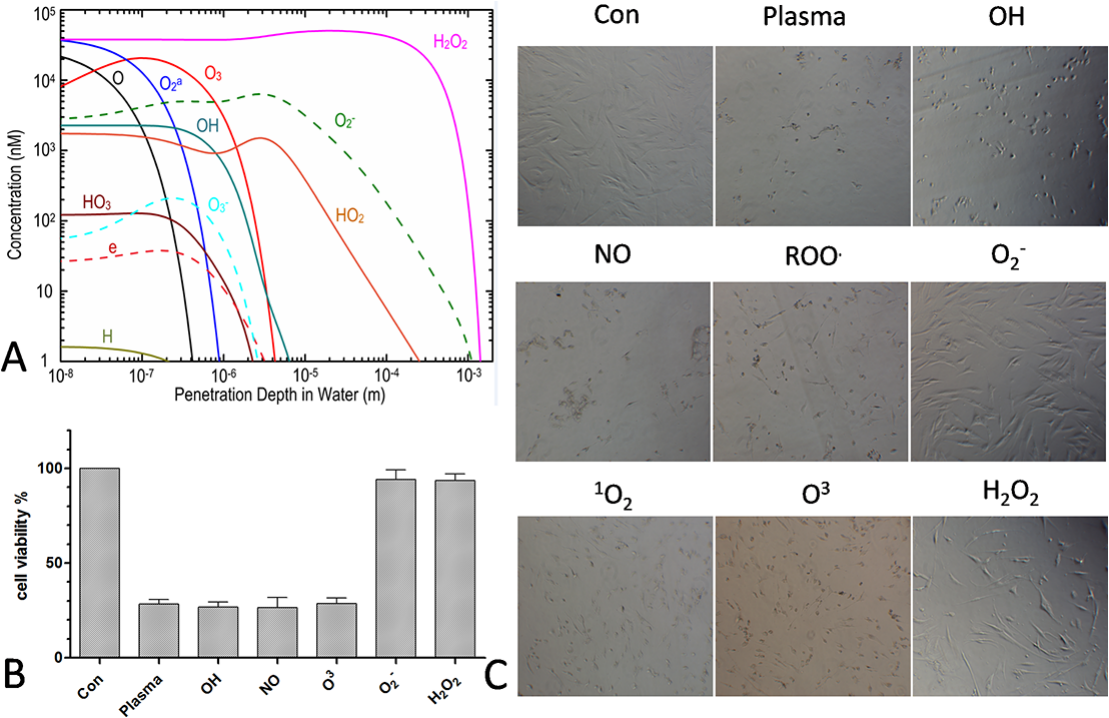
Plasma-induced cell death is reversed by reactive species scavengers. (A) Computer simulation of the distribution of various species in liquid. (B, C) MSC viability and morphological changes 24 h after treatment with He + H_**2**_O plasma for 60 s in the presence of various scavengers.

The boundary conditions and parameters of this model were described in our previous work [22]. Among the variety of radicals in the plasma gas phase, certain species were transferred further than others: while most disappeared or were converted into other species at a depth of 1 mm, O_2_^−^ and H_2_O_2_ reached depths of up to 2 mm (Fig 3A).

When scavengers for particular species were added in order to exclude the effect of the particular ROS and RNS generated during the process of plasma treatment, we first confirmed that these scavengers are non-toxic to cells at the working concentrations used in the assays (data not shown). The results showed that depletion of O_2_^−^ or H_2_O_2_, but not of other species such as OH, NO, ROO•, O^3^, ONOO^−^, or ^1^O_2_, annihilated the effects of plasma treatment, as seen by the changes in cell viability (Fig 3B) and cell morphology (Fig 3C). These results indicate that O_2_^−^ and H_2_O_2_ were the predominant species for induction of apoptosis generated by plasma treatment. Moreover, when 500–600 μl of culture medium were added to each well (yielding a depth of about 3 mm in a 24 well plate), the plasma treatment induced little or no cell death (data not shown).

**Fig 4:**
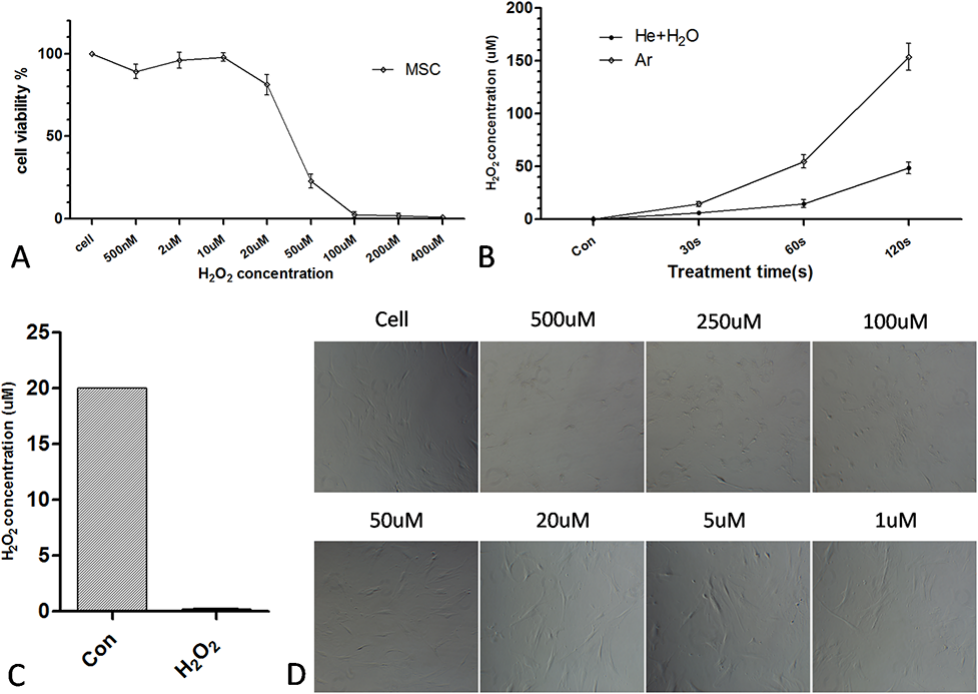
Contribution of H_2_O_2_ to plasma-induced cell death. (A) Viability of MSCs treated with different concentrations of H_**2**_O_**2**_ for 24 h. (B) H_**2**_O_**2**_ concentration measured by the hydrogen peroxide assay after He+H_**2**_O and Ar plasma treatment for the indicated times. (C) H_**2**_O_**2**_ concentration measured relative to the control (20 μM H_**2**_O_**2**_ solution) after adding H_**2**_O_**2**_ scavenger. (D) Morphological changes in MSCs treated with indicated H_**2**_O_**2**_ concentrations for 24 h.

To determine which of O_2_^−^ or H_2_O_2_ induces the greatest biological effect, cells treated with H_2_O_2_ with or without plasma were compared. H_2_O_2_ alone induced cell death at concentrations of 50–100 μM (Fig 4A). Only H_2_O_2_ concentrations > 100 μM caused morphological changes after 24 h (Fig 4D). H_2_O_2_ production was then measured after plasma treatment for different times. The concentration of H_2_O_2_ after a 60 s He + H_2_O plasma treatment was < 20 μM, suggesting that the H_2_O_2_ radical in itself is insufficient for inducing cell death (Fig 4B); H_2_O_2_ was completely abolished by treatment with an H_2_O_2_ scavenger, showing that scavenger could be a useful method to determine the effect of H_2_O_2_ (Fig 4C).

**Fig 5:**
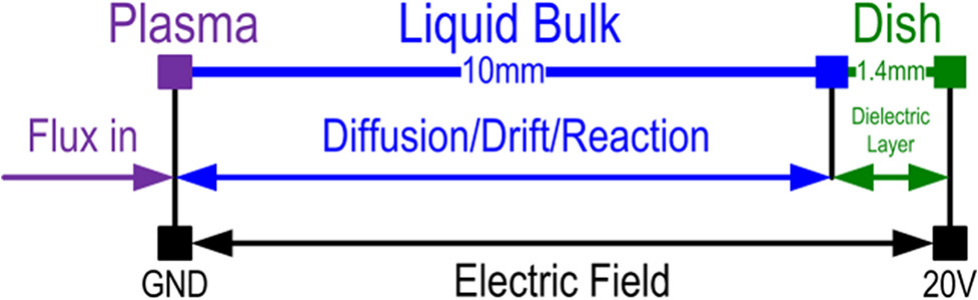
A schematic diagram for applying a bias voltage.

Because there is no O_2_^−^ reagent, cells treated with O_2_^−^ with or without plasma could not be compared. We therefore attempted to separate O_2_^−^ from H_2_O_2_ by applying a bias voltage, since O_2_^−^ is electronegative and is attracted by a positive voltage, as summarized in Fig 5.

The basic equations were the same as those previously described. Given the dielectric layer at the bottom, the charge conservation equation was included to calculate the effect of charge accumulation.

$$\left\{ \begin{array}{l} \frac{\partial \rho_{dish}}{\partial t}+\nabla •J_{dish}=0\\ J_{dish}=\sigma_{dish}E \end{array}\right.$$

The Poisson equation for the dielectric area was also applied.

$$\frac{\partial^{2}V}{\partial x^{2}}=\left\{ \begin{array}{l} -\frac{\sum \rho_{net}}{\varepsilon_{H_{2}O}}\\ -\frac{\rho_{dish}}{\varepsilon_{dish}} \end{array}\right.,E=-\frac{\partial V}{\partial x}$$

**Fig 6:**
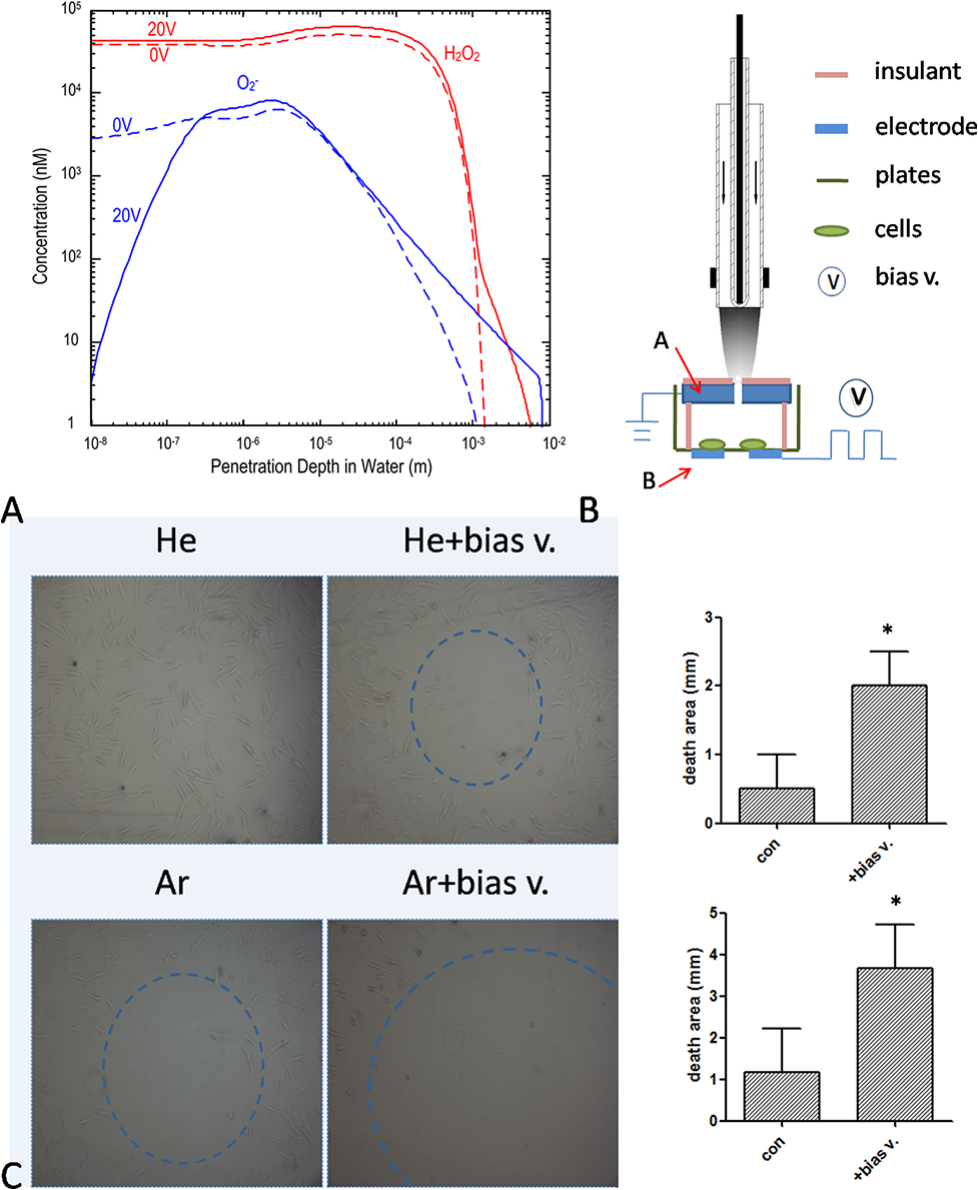
Contribution of O2− and H_2_O_2_ in plasma-induced cell death. (A) The O_**2**_^**−**^ and H_**2**_O_**2**_ distribution in liquid determined after applying a +20 V bias voltage by computer simulation. Broken and the solid lines represent concentrations of O_**2**_^**−**^ and H_**2**_O_**2**_, before and after voltage application, respectively,. (B) Experimental setup for application of bias voltage. A and B indicate cover node and bias voltage, respectively. (C) MSCs treated with He + H_**2**_O or Ar plasma for 60 s while applying a +20 V bias voltage. The histogram displays the diameter of the cell death area relative to the control. *P < 0.05.

The simulation showed that the penetration depth of O_2_^−^ was increased by applying a +20 V bias voltage, while that of H_2_O_2_ also increased slightly (Fig 6A). As a neutral species, H_2_O_2_ should theoretically be unaffected by a bias voltage. The slight increase observed for H_2_O_2_ was presumed to be caused by O_2_^−^. As stated in our previous work [22], only H_2_O_2_, HO_2_, and O_2_^−^ can exist at a depth of 1 mm; the reactions are as follows.

$$O_{2}^{-}+H^{+}\overset{97.9\%}{\to }HO_{2}$$

$$O_{2}^{-}+H_{2}O\overset{2.28\%}{\to }HO_{2}+OH^{-}$$

$$2HO_{2}\overset{86.2\%}{\to }H_{2}O_{2}+O_{2}$$

Applying a bias voltage increased the penetration depth of O_2_^−^, which may have been partly converted to H_2_O_2_, thereby increasing its concentration.

The experimental setup for testing the above simulation is shown in Fig 6B. Several sizes for the cover (A) and bias voltage node (B) were tested, and diameters of 1.5 and 5 mm, respectively, were ultimately used. Based on the simulation, the penetration of O_2_^−^ was predicted to be deeper and cover a broader area. Indeed, after applying a bias voltage of +20 V, the area of cell death cell was increased significantly by a 60 s He + H_2_O or Ar plasma treatment (Fig 6C). Furthermore, to exclude the effect of H_2_O_2_, a scavenger was added to the medium before the bias voltage was applied. Although O_2_^−^ penetration was still deeper and covered a larger area, in the absence of H_2_O_2_, the area of cell death was unchanged (data not shown). These results demonstrate that H_2_O_2_ and O_2_^−^ are the major reactive species that induce cell death. However, each species alone was insufficient to produce this effect, suggesting that a synergistic interaction occurs.

The suggestion of OH radical production being catalyzed by iron [23] has been experimentally validated by the discovery of several Fenton-like reactions [24–26]. In the iron-catalyzed Haber–Weiss reaction, OH is produced from H_2_O_2_ and O_2_^−^ radicals [26].

$$H_{2}O_{2}+O_{2}^{-}\overset{{\text{Fe}}^{2+}/{\text{Fe}}^{3+}}{\to }OH^{-}+∙OH+O_{2}$$

It is now widely acknowledged that the Haber–Weiss reaction does not occur in the absence of metal catalysts [27]; the reaction has been directly observed in the gas phase in the presence of iron [28].

We set out to determine whether the Haber–Weiss reaction occurs in a plasma-treated cell system. Findings from previous studies suggested that plasma treatment could provide both H_2_O_2_ and O_2_^−^ species at μM concentrations. Cells typically express several iron proteins such as ferroportin [29, 30], lactoferrin receptor [31], transferrin [32, 33], divalent metal transporter 1 [34, 35], and ferritin [36, 37], among others. These proteins could conceivably catalyze H_2_O_2_ and O_2_^−^ radicals into a highly reactive OH radical that has more potent biological effects.

**Fig 7:**
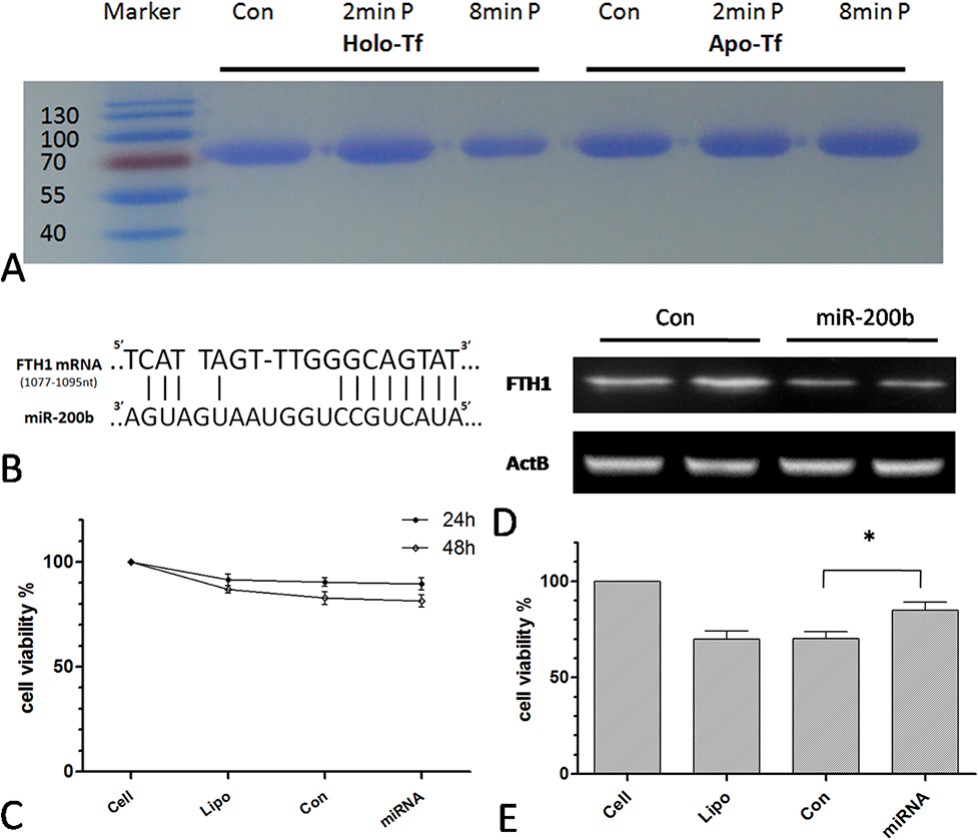
Decreased sensitivity to plasma by knockdown of iron protein expression. (A) Gel electrophoresis of holo- and apo-transferrin treated with Ar plasma for 2 or 8 min. (B) Sequences of miR-200b and the complementary site in the *FTH1* gene. (C) Viability of MSCs transfected with control miRNA or miR-200b for 24 or 48 h. (D) FTH1 expression in MSCs transfected for 48 h, as determined by western blotting. (E) Viability of transfected MSCs 24 h after 20 s of Ar plasma treatment. *P < 0.05. Lipo indicates that only the transfection agent (Lipofectamine 2000) was added to cells.

To test this hypothesis, transferrin—which is known as holo- or apo-transferrin depending on whether it is or is not bound to iron, respectively—was examined. Both forms of the protein (0.1 mg/ml in 300 μl PBS solution) were treated with Ar plasma and protein degradation was assessed by gel electrophoresis. After an 8 min plasma treatment, holo- but not apo-transferrin showed significant degradation (Fig 7A), suggesting that the presence of iron resulted in the catalysis of the Haber–Weiss reaction, thereby enhancing the effects of the plasma. It has been reported that the superoxide anion may also induces the release of ferrous iron from transferrin[38], further facilitating the Haber-Weiss reaction and consequent OH formation. Ferritin expression was then knocked down with miR-200b [39] and cells were examined for their sensitivity to plasma treatment; the sequences of miR-200b and the complementary site in the *FTH1* gene are shown in Fig 7B. Cell viability was decreased by knockdown of *FTH1* gene expression (Fig 7C). After 24 h of transfection, cells were treated with Ar or He + H_2_O plasma (data not shown) for 20 s followed by a 24 h incubation. The downregulation of ferritin expression by miR-200b as compared to the control transfection was confirmed by western blotting (Fig 7D), and was associated with a decrease in cell sensitivity to Ar plasma treatment (Fig 7E). These data suggest that iron proteins act as catalysts that transform H_2_O_2_ and O_2_^−^ into the highly reactive OH radical, which then induces cell death.

**Fig 8:**
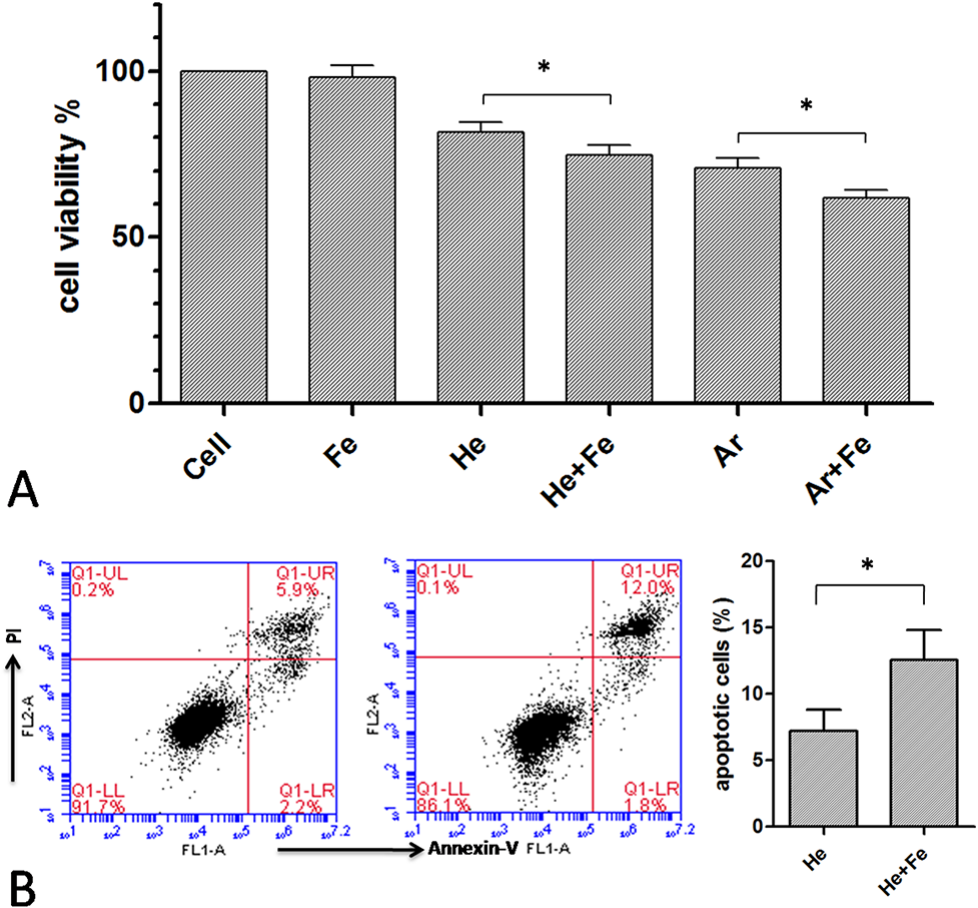
Iron enhances plasma-induced cell death. (A) Viability and (B) apoptosis of LP-1 cells 24 h after a 20-s He + H_**2**_O or Ar plasma treatment in the presence of Fe(III)-EDTA. *P < 0.05.

Iron was added in the form of Fe(III)-EDTA to the plasma-treated cell system to enhance OH production and consequently cell death. To circumvent the possibility that the penetration of iron-catalyzed OH would not be sufficiently deep to affect MSCs given their adherence, an LP-1 cell suspension [16] was used in this experiment. To increase the probability of interaction between OH and LP-1 cells, the cells were treated with He + H_2_O or Ar plasma for 20 s at a relatively high concentration (5 × 10^6^/ml). Fe(III)-EDTA (1 μM) had no effect on cell viability by itself, but enhanced plasma-induced cell death (Fig 8A). This was confirmed by flow cytometry in which apoptotic cells were visualized by annexin V-FITC and PI staining (Fig 8B).

**Fig 9:**
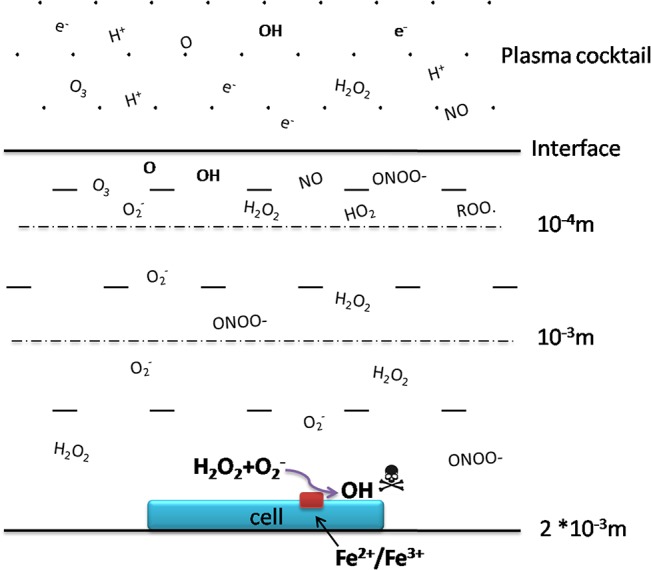
Model for a plasma-treated cell system and the mechanism of *in situ* OH generation.

CAP can produce various reactive species such as the OH radical, which is the most highly reactive and can damage most macromolecules including DNA, proteins, lipids, or polysaccharides. OH is 100 fold more potent than H_2_O_2_ and O_2_^−^ and can affect molecules located a few nanometers from its site of generation [40]. However, given its high reactivity, OH has an extremely short half-life (in the nanosecond range). Thus, although CAP can generate OH radicals at μM concentrations, these species are unlikely to reach cells and cause damage to biomolecules; this can only be achieved by long-lived H_2_O_2_ species [41, 42]. As such, it is debatable whether plasma medicine constitutes nothing more than H_2_O_2_ treatment. The present study showed for the first time that two major reactive species—H_2_O_2_ and O_2_^−^—can penetrate to a depth that is sufficient to reach cells; we also propose a novel model of *in situ* generation of OH, which acts as the final effector causing cellular damage (Fig 9). This model can explain why treating cells with H_2_O_2_ or O_2_^−^ scavengers can reverse the biological effects of plasma treatment while treatment with an OH scavenger had little effect. The model also highlights the synergy between H_2_O_2_ and O_2_^−^ at relatively low concentrations (compared to their respective lethal doses). Under physiological conditions, H_2_O_2_ and O_2_^−^ concentrations are rarely low—estimates for O_2_^−^ within the mitochondrial matrix are in the range 10–200 pM [43]—and therefore the iron-catalyzed Haber-Weiss reaction has a negligible effect on cells. However, in a plasma-treated cell system, the plasma provides high levels of both H_2_O_2_ and O_2_^−^, with iron-catalyzed OH production thereby greatly influencing cell viability. These findings provide a basis for achieving optimal biological effects by plasma treatment using this *in situ* mechanism for OH generation.

## Conclusion

In conclusion, the present study demonstrated for the first time by biological experiments that H_2_O_2_ and O_2_^−^ are the two major reactive species that are produced by plasma treatment. Given that the concentration of either species is insufficient to induce cell death, we propose that the OH radical generated *in situ* of the cells by the Haber–Weiss reaction ultimately causes cell damage and death.

## Supporting Information

S1 Fig Experimental setup for plasma treatment. The apparatus consisted of a gas flow controller, high-voltage power supply, oscilloscope, and plasma jet.
(TIF)
