# Supplementary figures and images for: In Situ OH Generation from O2 − and H2O2 Plays a Critical Role in Plasma-Induced Cell Death
Source: PLoS One. 2015 Jun 5;10(6):e0128205. doi: 10.1371/journal.pone.0128205 (PMC4457797; doi:10.1371/journal.pone.0128205)

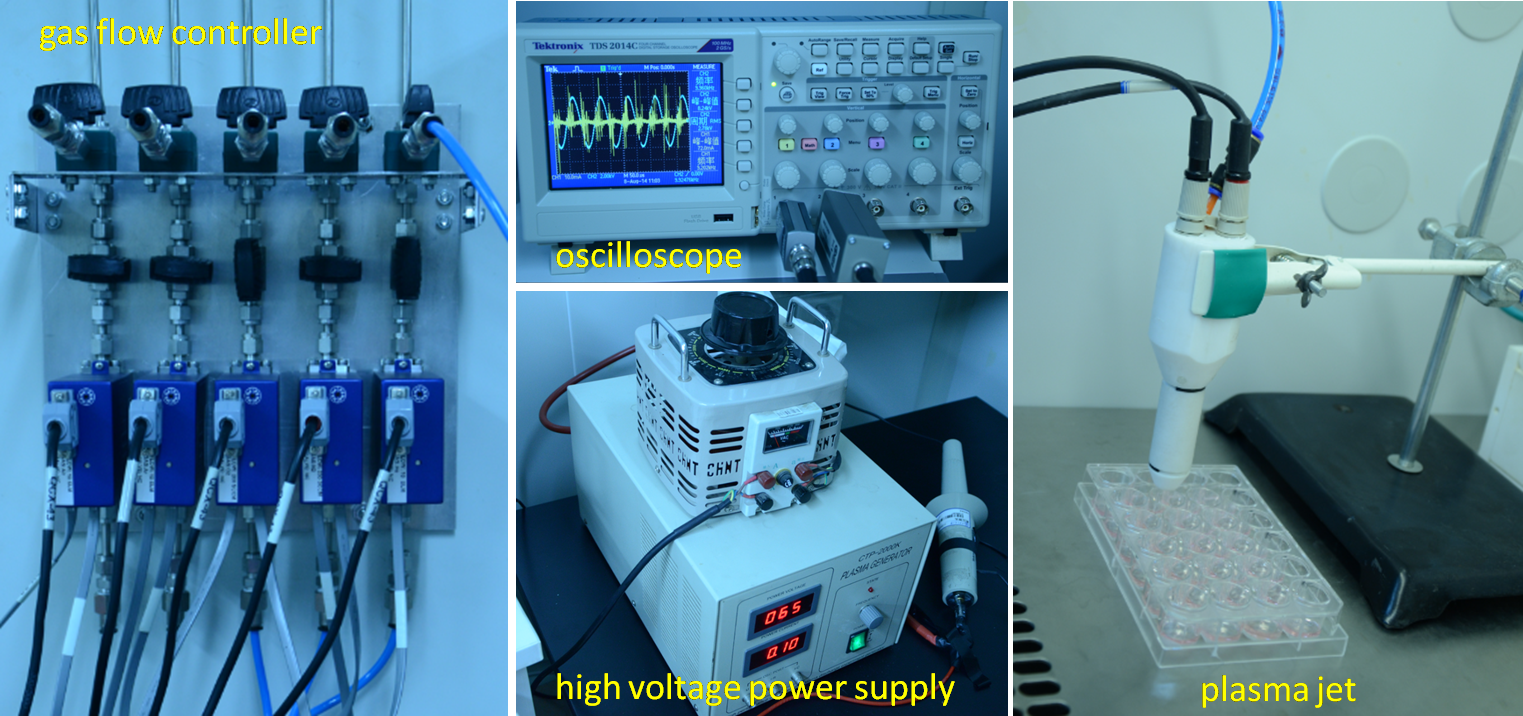

Supplement: S1 Fig — The apparatus consisted of a gas flow controller, high-voltage power supply, oscilloscope, and plasma jet. (TIF) [file pone.0128205.s001.tif]
